# A novel efficient multi-walled carbon nanotubes/gibberellic acid composite for enhancement vase life and quality of *Rosa hybrida* cv. ‘Moonstone’

**DOI:** 10.1186/s12870-024-04925-9

**Published:** 2024-04-03

**Authors:** Hany M. El-Naggar, Shimaa M. Ali, Amira R. Osman

**Affiliations:** 1 Department of Floriculture, Faculty of Agriculture, Alexandria University (El-Shatby), Alexandria, 21545 Egypt; 2 Department of Chemistry, Faculty of Science, Cairo University, Giza, 12613 Egypt; 3 Department of Horticulture, Faculty of Agriculture, Damanhour University, Damanhour, Beheira, 22516 Egypt

**Keywords:** Antioxidant defense, Carbon nanotubes, Fresh flower life extension, Nanomaterials, Plant growth regulator, Preservation solution

## Abstract

The postharvest life of cut flowers is limited, which is a major challenge and varies greatly depending on plant varieties, cut flower stage, flower length of the harvested shoots, and storage conditions including postharvest treatments. As a result, improving the vase life and quality of cut flowers in regulating postharvest characteristics and overcoming these challenges is critical to the horticulture business. Novel engineered nanocomposites were created and tested for possible impacts on flower bud opening, postharvest life extension, longevity regulation, and preservation and enhancement of the strength and appearance of cut flowers. The experiment was conducted as a factorial experiment using a completely randomized design (CRD) with two factors. The first factor was two holding solutions (without or with sucrose at 20 gL^−1^). The second factor was 12 pulsing treatments for 24 h; distilled water as a control, 75 ppm GA_3_, multi-walled carbon nanotubes MWCNTs at 10, 20, 30, 40, and 50 ppm, and MWCNTs (10, 20, 30, 40, and 50 ppm)/GA_3_ (75 ppm) composites; each treatment had 3 replicates, for a total of 72 experimental units. In the present study, gibberellic acid (GA_3_) was synthesized in functionalized (MWCNT/GA_3_ composites) as a novel antisenescence agent, and their effect on the vase life quality of cut rose flowers *Rosa hybrida* cv. ‘Moonstone’ was compared by assaying several parameters critical for vase life. The adsorption of GA_3_ on MWCNTs was proven by performing FTIR spectroscopy which ensures that the formation of the MWCNTs/GA3 composite preserves the nanostructure and was examined by high-resolution transmission electron microscopy (HR-TEM). The results revealed that sucrose in the holding solution showed a significant increase in fresh weight, flower diameter, and vase life by 10.5, 10.6, and 3.3% respectively. Applying sucrose with MWCNTs 20 ppm/GA_3_ 75 ppm composites or MWCNTs 20 ppm alone, was critical for the significant increase in flower opening by 39.7 and 28.7%, and longevity by 34.4 and 23.2%, respectively, and significantly increased chlorophyll a, b, total chlorophyll, anthocyanin, total phenolic content, and 2,2-Diphenyl-1-picrylhydrazyl scavenging activity as compared to the control.

**Supplementary Information:**

The online version contains supplementary material available at 10.1186/s12870-024-04925-9.

## Introduction

The main challenge with cut flowers is their limited postharvest life (approximately 6-15 days), which greatly depends on variety and storage circumstances [1] since they are very susceptible to mechanical damage and ethylene. Stem blockage caused by microbial infection (bacteria) at the cut end of the stem, vascular occlusion, and physiological damage are often the main causes of vase life loss in cut flowers. Additionally, ethylene biosynthesis, water imbalance, and the depletion of food reserves (carbohydrates) all affect postharvest life [2]. As a result, vase life can be extended by soaking cut stems in a preservative solution containing nanomaterials or nanoparticles with precise dispersion, such as nanosilver, which has been utilized for some cut flowers, such as carnations, and establishing an optimum environment [3] and chitosan nanoparticles on indices vase life on rosa [4].

A rose is an attractive shrub of the Rosaceae family. Roses are the most widely used cut flower in the global floral market [5]. They are used as flower or ornament arrangements and interior decorations because of their beautiful and delicious character, as well as their fragrances. With their gleaming colors, forms, sizes, and perfumes, the roses offer a spectacular formal display. In the floriculture industry, increasing the durability and quality of cut flowers is a top objective. Long vase life and appropriate opening of the flower buds are important quality requirements for consumers [6].

Depending on the characteristics needed for the desired application, nanomaterials (NMs) and nanoparticles (NPs) can take on a wide variety of shapes, sizes, dimensions, origins, and compositions [7]. They come in shapes ranging from 1 to 100 nm, including tubular, spherical, hollow, flat, and irregular shapes [8]. NPs contain unique and size-related physio-chemical characteristics that distinguish them from larger materials [9]. To reduce the amount of horticultural product waste, nanotechnology also contributes to the postharvest life extension of many horticultural products in a variety of ways, including the creation of new, creative preservative materials (novel nanocomposites), regulating the longevity of cut flower postharvest, flower bud opening and preserving and enhancing cut flower strength and appearance [10].

NMs in combination with plant growth regulators (PGRs) are mostly utilized to modulate hormone levels to maximize the production value. Furthermore, NMs aid in PGR absorption and distribution within the plant [7].

Carbon-based nanomaterials congregate in aqueous solutions or on the surface of tissues because they are hydrophobic, insoluble, or have limited dispersive capacity [11]. This trait, on the other hand, boosts their ability to interact with a wide range of chemical compounds and plant tissue, resulting in increased biological activity [12]. Their impact is generally proportional to the nanomaterial size, concentration, and solubility [13]. Multiple strategies (both covalent and noncovalent) for achieving homogenous nanotube dispersions have been proposed. Surfactants such as anionic, cationic, nonionic, and polymer wrapping are effective dispersion agents of nanotubes [14]. The primary emphasis of the nanomaterial family is the MWCNTs, which are made up of multiple thin layers of coiled graphene sheets that have sp^2^ hybridization; because of their outstanding qualities, they have demonstrated amazing performance in a wide range of applications [15, 16]. After achieving homogenous MWCNTs, dispersions have been proposed to diffuse into vase water and travel through stem vessels to the leaves via transpiration [17–19]. Nanocomposites can be used securely and successfully for *Alstroemeria* cut flowers in vase solutions, MWCNTs or in a mixture with other preservatives, enhancing their vase life with quality compared to the control [10].

PGRs and plant hormones have the same chemical structure and biological effects; the only difference is how they are derived. At very low concentrations, they govern plant growth and development [20]. Gibberellins (GA) are one of five major classes of endogenous plant hormones that boost certain physiological or biochemical pathways in plants; GA_3_ is believed to be a plant growth regulator that delays senescence [21]. The application of GA_3_ had a substantial effect on the vase life, total chlorophyll, and carotene content of leaves in *Solidago canadensis* L.cv. “Tara” [22]. GA_3_ pulsing improves flower petal water content, and solution uptake increases vase life and other qualitative attributes of cut stems compared with the control and delays the bent neck of cut rose [23].

The carbohydrate contents and dry weight of cut flower stems may estimate the possible vase life of bloom at a given temperature, as carbohydrates, primarily sugars, gradually decrease during respiration. Higher levels of endogenous carbohydrates reduce ethylene sensitivity but do not reduce ethylene production [24].

Sucrose is frequently used as an external pulsing or holding treatment for cut flowers. It maintains dry matter content and level respiration, improves water balance in the plant, and is effective in the management of stomata, which decreases water evaporation and thus increases the vase life quality of Carnation and Rosa, respectively cut flowers, marketability and commercial value [25, 26].

The purpose of this study was to determine and compare the efficacy of pulsing treatments of MWCNTs, gibberellic acid, and MWCNTs/GA_3_ composites, a novel nanocomposite, as anti-senescence agents employed at different concentrations. The holding solutions were used with or without sucrose as a carbohydrate supply resource to improve some qualitative and physiological characteristics of cut rose flowers (*Rosa hybrida* cv. ‘Moonstone’) in the regulation of postharvest attributes, some parameters, characteristics, photosynthetic pigments, antioxidant activity, and quantification of the total phenolic content were measured.

## Materials and methods

### Materials

GA_3_ (Sigma‒Aldrich Bangalore India), MWCNTs, D $\times$L 110-170 nm $\times$ 5-9 μm, purity >90% carbon basis, Aldrich, absolute ethanol (Sigma‒Aldrich).

### Preparation of 75 ppm GA_3_ solution

The GA_3_ stock solution was prepared by weighing 0.075 g of GA_3_ and dissolving it in 100 ml of absolute ethanol. Then, it was diluted to 1 L with distilled water.

### Preparation of the MWCNT series and MWCNT/GA_3_ composite series

Two series of MWCNT aqueous solutions and MWCNTs in 75 ppm GA_3_ solutions with varied concentrations, 10, 20, 30, 40, and 50 ppm, were made by weighing the needed quantity of MWCNTs in 100 ml of distilled water and 75 ppm GA_3_ solution. To guarantee the homogeneity of mixing and formation of the nanocomposite, the solution was sonicated for 1 h in an ultrasonic bath (Branson® ultrasonic bath, 115 Vac, 60 Hz).

### Plant material, treatments, and experimental design

Uniform cut rose flowers *Rosa hybrida* cv. ‘Moonstone’ based on the diameter in the showing color stage (stage 2) [27], and the harvested shoots had a length of approximately 0.7 m. These were harvested early in the morning (07:00-9:00 AM) from a commercial greenhouse in Giza governorate, Egypt, coordinates (30° 6’ 3.78” N, 31° 7’ 0.53” E). The flowers were preserved using ice gel bags within an ice box and transferred within 3 h after harvest to the laboratory of the Floriculture Department, Faculty of Agriculture, Alexandria University, where the experiment was performed through April and May of 2023. All thorns and the lower leaves of the cut rose were removed gently, the basal 20 cm of the stem, as soon as the flowers arrived at the lab. Prior to the treatments, all rose stems were recut to 0.6 m in length under distilled water (to prevent cavitation of xylem vessels that were opened by cutting), and then each flower was placed individually in a glass tissue culture tube (2.5 cm in diameter by 15 cm height) as a vase containing 50 mL of either pulsing or holding solution. All tube openings were covered with aluminum foil paper to reduce both contamination and evaporation.

Two holding solutions, with or with no sucrose (S and NS, respectively) at 20 g/L, were used supplemented with MWCNTs alone, MWCNTs combined with 75 ppm GA_3_ as a novel nano anti-senescence agent composite and GA_3_ at 75 ppm alone (MWCNTs, MWCNTs/GA_3_ 75 composites and GA_3_ 75, respectively) employed at different concentrations with S as holding solution as follow (T1= Control; distilled water, T2= MWCNTs 10, T3= MWCNTs 20, T4= MWCNTs 30, T5= MWCNTs 40, T6= MWCNTs 50, T7= GA_3_ 75, T8= MWCNTs 10/GA_3_ 75 composites, T9= MWCNTs 20/GA_3_ 75 composites, T10= MWCNTs 30/GA_3_ 75 composites, T11= MWCNTs 40/GA_3_ 75 composites, and T12= MWCNTs 50/GA_3_ 75 composites ppm) the same concentrations were used without S, a total of 24 treatments, all were used as pulsing treatments for 24 h, silver nitrate AgNO_3_ (Macsen Laboratories Rajasthan, India) at 10 ppm was added to both holding solutions to eliminate microorganisms growth [28]. The initial pH of the holding and the pulsing solutions were 7 - 7.2 and 6.2 - 6.5 respectively, both were adjusted by drops of citric acid to 5.8 ± 0.1 using a pH meter (211 Hanna Instruments, Cluj-Napoca, Romania). The cut flower tubes were randomly placed in a ventilated laboratory at 24 °C ± 2 °C, relative humidity 65 ± 5% RH, under cool white, fluorescent lamps (55–56 mol/m^2^/S), placed 40 cm above the flowers with a 12 h light/dark photoperiod per day until the end of the experiment. Flowers were evaluated for their physiological and biochemical traits during the experiment. Most analyses were recorded when the maximum flower diameter was reached.

### Physiological and biochemical trait assays

#### Flower fresh weight

The fresh weight of the flowers was measured with an analytical balance and a digital scale with an accuracy of 0.001 g (Setra BL-410 precision balance USA) each week during the vase period, and the differences in fresh weight of the flowers with the initial values were calculated. Then the maximum flower fresh weight of the flowers was recorded at the maximum diameter of the flower.

#### Flower diameter

The flower diameter was measured using a Vernier caliper (Poland) each week during the vase period, and then the maximum flower diameter was recorded and photographed with a Stanley tape measure for effective visualization [29].

#### Vase life

Vase life was measured as the days from the first day of the vase in stage 2 in which the roses started different treatments until the roses lost their ornamental appeal, as described by rose wilting, withering, and discoloration of the rose, the petals becoming wrinkled with a bowed neck as the beginning of the aging process [29]. The following were the flower opening indices: Stage 0, unopened bud; Stage 1, partially opened bud; Stage 2, completely opened bud; Stage 3, partially opened flower; Stage 4, fully opened flower without anther appearance; and Stage 5, fully opened flower with anther appearance (yellow) described by Jishi et al. [30].

#### Photosynthetic pigments; chlorophyll a, chlorophyll b, total chlorophyll, and carotenoid contents of rose flower fresh leaves

The decreases in chlorophyll concentrations are related to leaf yellowing, which is a sign of termination of vase life [27]. As a result, the photosynthetic pigments of the leaves were evaluated under various treatments at the maximum flower diameter stage [31]. Fresh leaf samples (0.1 g) were washed and incubated overnight at a cool temperature (4-5 °C) in 5 ml of N,N-dimethyl formamide solution. A spectrophotometer (Unico W49376 Spectrophotometer 1200, China) was used to quantify chlorophyll a, b, total chlorophyll, and carotenoids at 647, 663, and 470 nm [32] formulae were used to compute chlorophylls and carotenoids (mg/g fresh weight) as follows:

- Chl. a =12.70 A_663_ – 2.79 A_647_
- Chl. b =20.76 A_647_ – 4.62 A_663_
- Total Chls =17.90 A_647_ + 8.08 A_663_
- Carotenoids = [1000 A_470_ – (3.72 chl. a - 104 chl. b)]/229

#### Quantification of the total phenolic compounds (TPC) of rose leaves

The total phenolic content of a methanolic extract of rose leaves was assessed using the Folin-Ciocalteu technique. The methanol extract of the samples (0.2 ml, 100.0 gm/L) was mixed in distilled water with a volume of 2.0 ml of diluted Folin-Ciocalteu reagent (1:10). After 5 min, a saturated NaHCO_3_ solution (1.5 ml, 60 g/L distilled water) was applied. All combinations were allowed to remain at room temperature for 90 min before the absorbance was measured using a spectrophotometer at 725 nm (Unico W49376 spectrophotometer 1200; Shanghai, China). Milligrams of gallic acid equivalents (GAE) per gram of dried extract were used to calculate the total phenolic content [33, 34].

#### DPPH radical scavenging activity analysis

The plant extract was made by separating the leaves and drying them at room temperature in the dark. Then, using a homogenizer, 2 g of freshly crushed dry leaves were extracted with 100 mL of 80% methanol. The mixtures were centrifuged at 5000 rpm, and the liquid extracts were frozen at -20 °C for further analysis. Spectrophotometry was used to analyze the extract’s capacity to scavenge radicals against stable DPPH. To give null corrections, a blank composed of aliquots of 3 mL of 90% aqueous methanol without DPPH and the solvent extract was used in the spectrophotometer. When DPPH interacts with an antioxidant, it releases hydrogen and becomes reduced. The color shift occurred at 517 nm, transitioning from deep violet to brilliant yellow. A total of 1.5 mL of methanolic leaf extract was collected for each sample, and 1.5 ml of 0.1 mM DPPH solution produced in 90% methanol was added. Before use, the combination was completely mixed and stored in the dark at 4 °C, and the absorbance of the resulting solution was measured at 517 nm [35, 36].

$$\mathrm{Scavenging}\;\mathrm{activity}\;(\%)\;=\;(1-\mathrm{absorbance}\;\mathrm{of}\;\mathrm{sample}\;\mathrm{at}\;517\;\mathrm{nm}/\mathrm{absorbance}\;\mathrm{of}\;\mathrm{control}\;\mathrm{at}\;517\;\mathrm{nm})\;\times100$$

$$\mathrm{Antiradical}\;\mathrm{activity}\;(\mathrm{DPPH})\;(\%)\;=\;[(\mathrm{absorbance}\;\mathrm{of}\;\mathrm{control}\;-\;\mathrm{absorbance}\;\mathrm{of}\;\mathrm{sample})/\mathrm{absorbance}\;\mathrm{of}\;\mathrm{control}]\;\times\;100\;$$

#### Anthocyanin content in fresh rose petals

Total anthocyanin concentrations in rose petal extract were determined after 30 min of incubation in acidified methanol (methanol + 1% HCl). For 10 min, the extracts were centrifuged at 10,000 rpm. The sample was prepared in 5 ml increments and diluted in 0.4 M (pH 4.5) sodium acetate buffer and 0.025 M (pH 1.0) potassium chloride buffer (5 ml each). After a 15-minute incubation period at room temperature, the absorbance at 520 and 700 nm was measured using a spectrophotometer (Unico W49376 Spectrophotometer 1200, Shanghai, China), and the total anthocyanin contents were expressed as milligrams per 100 g of fresh weight (FW) [37, 38].

$$\mathrm{Total}\;\mathrm{anthocyanin}\;\mathrm{concentrations}\;(\mathrm{mg}/100\;\mathrm g\;\mathrm{FW})\;=\;\mathrm A\;\mathrm \times\;\mathrm{MW}\;\mathrm \times\;\mathrm D\;\mathrm \times\;1000/\mathrm\varepsilon$$

$$\mathrm A\;(\mathrm{absorbance}\;\mathrm{value})\;=\;\lbrack(\mathrm A510\;\mathrm{nm}\;-\;\mathrm A700\;\mathrm{nm})\;\mathrm{pH}\;1.0\;\mathrm h\;-\;(\mathrm A510\;\mathrm{nm}\;-\mathrm A700\;\mathrm{nm})\;\mathrm{pH}\;4.5\rbrack$$

$$\mathrm{MW}\;(\mathrm{molecular}\;\mathrm{weight}\;\mathrm{of}\;\mathrm{cyanidin}-3-\mathrm{Oglucoside})\;=\;449.2$$

$$\mathrm D=\mathrm{dilution\;factor}$$

$$\upvarepsilon\;(\mathrm{molar}\;\mathrm{absorptivity}\;\mathrm{coefficient}\;\mathrm{of}\;\mathrm{cyanidin}-3-\mathrm{Oglucoside})\;=\;26,900.$$

### Statistical analysis

The experiment conducted for this study was set up as a factorial experiment of treatments (2 holding solution × 12 pulsing treatment = 24 treatments; each treatment had 3 replicates, for a total of 72 experimental units). According to Snedecor and William [39], the experimental design was a completely randomized design (CRD). Using SAS software [40], all the data gathered were subjected to analysis of variance (ANOVA) to compare the various treatments. Tukey’s test was used to compare mean values for several comparison ranges of means at the LSD_0.05_ level.

## Results

### The structural and surface characterization of the MWCNTs/GA_3_ composite

#### Fourier transform infrared (FTIR) spectroscopy

**Fig. 1:**
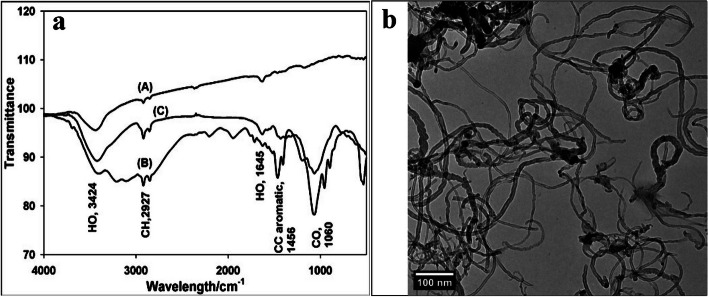
**a** The Fourier transform infrared (FTIR) spectra of (A) MWCNTs, (B) GA_3_, and (C) MWCNTs/GA_3_composite. **b** High resolution-transmission electron microscopy (HR-TEM) image of MWCNTs/GA_3_ composite

The adsorption of GA_3_ on MWCNTs was proven by performing FTIR spectroscopy (FTIR-84005SHIMADZU) of bare MWCNTs, bare GA_3_, and the MWCNT/GA_3_ composite, as shown in Fig. 1a. The MWCNT/GA_3_ composite sample for FTIR analysis was prepared by adding MWCNTs (20 ppm) to a 75 ppm GA_3_ solution and sonicating for 2 h. The composite was then filtered and dried at 60 °C for 1 h. The characteristic GA_3_ bands appeared clearly in the FTIR spectrum of the MWCNT/GA_3_ composite. Bands at 3424 and 1645 cm^-1^ are assigned to phenolic, alcoholic, and carboxylic O-H stretching vibrations, and a band at 2927 cm^-1^ is assigned to C-H stretching. Bands at 1456 and 1060 cm^-1^ correspond to C-C stretching in the aromatic ring, and C-O stretching, respectively [41] and [42].

The surface morphology of the MWCNTs/GA_3_ composite is examined by high-resolution transmission electron microscopy (HR-TEM) (Talos F200i S/TEM), as shown in Fig. 1b. The hollow morphology of MWCNTs can be identified even after GA_3_ adsorption, which ensures that the formation of the MWCNTs/GA_3_ composite preserves the nanostructure.

#### Effect of holding solution with S or NS and different pulsing solutions on morphological flower quality fresh weight, flower diameter, and vase life of cut rose

**Fig. 2:**
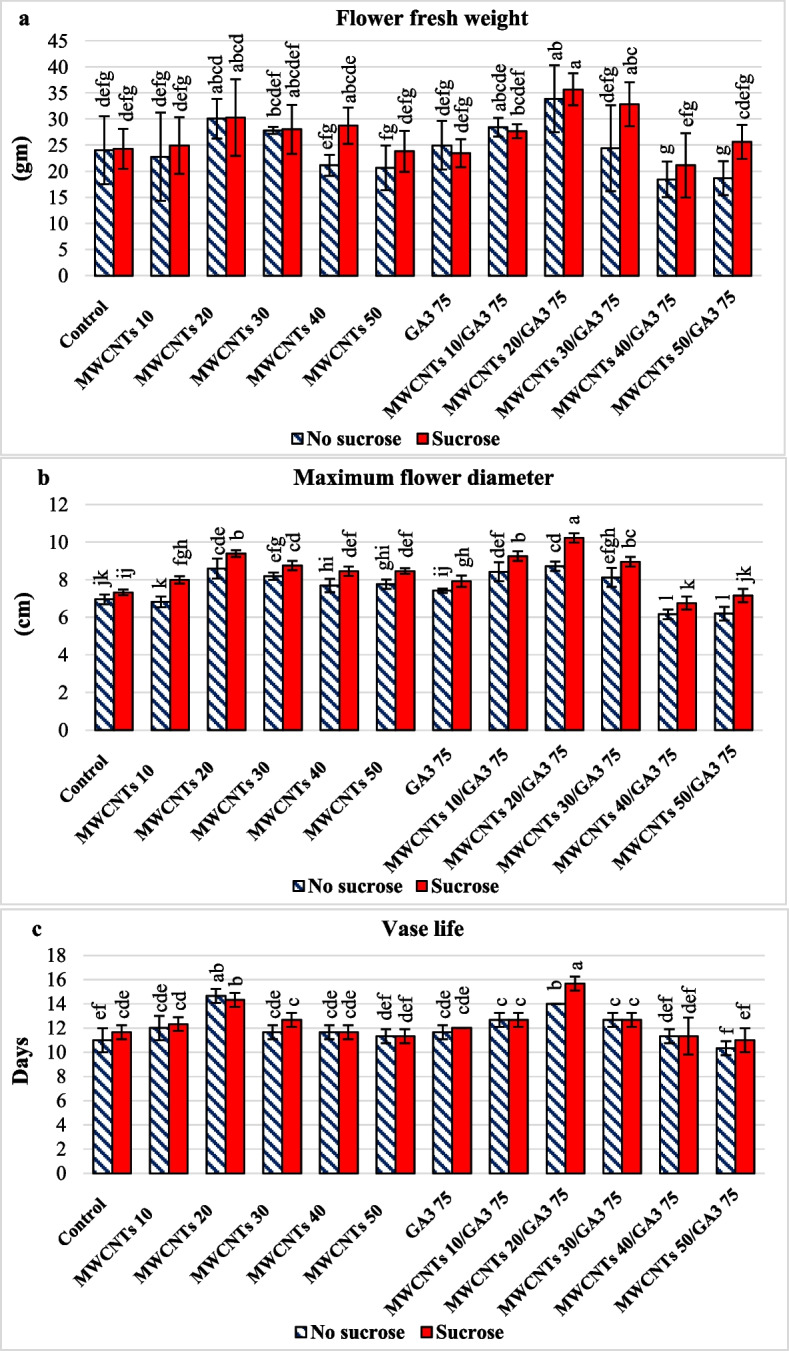
Cut rose moonstone morphological flower quality fresh weight (**a**), flower diameter (**b**), and vase life (**c**) as a function of holding solution; with sucrose (S) or no sucrose (NS) and different pulsing solutions. The interactions are expressed as the means ± the standard error (SE). Bars with the same lowercase letters are not significantly different at the *P* < 0.05 level; statistics are provided in Additional file 1: Table S1

Sucrose in the holding solution generally showed a significant increase in fresh weight (27.2 gm), maximum flower diameter (8.3 cm), and vase life (12.4 days) of cut rose (Table 1) and (Additional file 1: Table S1). In all cases, the main effect of the MWCNTs 20 ppm/GA_3_ 75 ppm composite was the optimal concentration as a pulsing solution and promoted positive significance on fresh weight 34.8 gm, maximum flower diameter 9.4 cm, and vase life 14.8 days of cut rose (Table 1) and (Additional file 1: Table S1). For MWCNTs at 20 ppm, alone in the pulsing solution promoted a significant increase in fresh weight of 30.2 gm, maximum flower diameter of 9.0 cm, and vase life of 14.5 days of cut rose (Table 1) and (Additional file 1: Table S1) and (Fig. 2). The largest significant effect on morphological flower quality; fresh weight 35.7 gm, maximum flower diameter 10.2 cm, and vase life 15.6 days of cut rose, respectively were detected on the interaction between S in holding solution with MWCNTs 20 ppm/GA_3_ 75 ppm composite in the novel anti-senescence agent engineered nanocomposites as a pulsing solution if compared with the control and the other treatments, as illustrated in Additional file 1: Table S1 and (Fig. 2a, b, c) while the lowest fresh weight 18.7 and 18.5 gm, minimum flower diameter 6.2 and 6.1 cm and vase life 10.3 and 11.3 days of cut rose, respectively were recorded in the interaction between NS in holding solution with MWCNTs 50 or 40 ppm/GA_3_ 75 ppm composite, respectively in pulsing solution as presented in (Additional file 1: Table S1).

**Table 1:** The comparison of the main effect of holding solution (HS) with No Sucrose (NS) or with Sucrose (S) and pulsing solution (PS) with novel anti-senescence agent-engineered nanocomposites on the morpho-physiological and phytochemical characteristics of cut fresh *Rosa hybrida* cv. Moonstone

	FW (gm)	FD (cm)	VL (days)	Chlor. a (mg/g FW)	Chlor. b (mg/g FW)	Total Chlor. (mg/g FW)	Carotenoid (mg/g FW)	Anthocyanin (mg/100 g FW)	TPC (mg GAE g -^1^)	DPPH (%)
**Main effect of holding solution (HS) with Sucrose (S) or No Sucrose (NS)**										
** No Sucrose (NS)**	24.6 B	7.5 B	12.0 B	1.1 B	0.6 B	1.8 B	0.52 B	10.6 B	17.2 B	47.1 B
** Sucrose (S) 20 gl** ^**-1**^	27.2 A	8.3 A	12.4 A	1.2 A	0.7 A	2.0 A	0.57 A	12.8 A	18.1 A	50.3 A
** LSD**_**0.05 (HS)**_	2.249	0.144	0.326	0.083	0.049	0.132	0.044	0.509	0.813	1.589
**Main effect of pulsing solution (PS) with novel anti-senescence agent-engineered nanocomposites**										
** Distilled water (Cont.)**	24.2 BC	7.1 EF	11.3 BC	1.1 B	0.7 B	1.8 BC	0.46 C	8.0 HI	14.5 CD	40.4 CDE
** MWCNTs 10 ppm**	23.9 BC	7.4 E	12.1 B	1.2 B	0.7 B	1.8 BC	0.47 BC	11.3 EF	13.5 CD	43.6 BCD
** MWCNTs 20 ppm**	30.2 AB	9.0 AB	14.5 A	1.3 B	0.8 B	2.2 B	0.51 BC	18.9 A	23.0 AB	75.3 A
** MWCNTs 30 ppm**	28.0 A-C	8.4 BC	12.1 B	1.2 B	0.7 B	2.1 BC	0.56 A-C	15.3 BC	23.0 AB	46.4 BC
** MWCNTs 40 ppm**	25.0 BC	8.0 CD	11.6 BC	1.1 B	0.7 B	1.8 BC	0.69 A	10.7 FG	15.0 CD	48.3 B
** MWCNTs 50 ppm**	22.2 BC	8.1 CD	11.3 BC	1.0 B	0.6 B	1.6 C	0.65 AB	9.0 GH	13.6 CD	44.1 BCD
** GA**_**3**_ **75 ppm**	24.2 BC	7.6 DE	11.8 BC	1.0 B	0.6 B	1.6 C	0.54 A-C	11.3 EF	16.9 C	38.6 DE
** MWCNTs 10/GA**_**3**_ **75 ppm composites**	28.1 A-C	8.8 B	12.6 B	1.0 B	0.6 B	1.8 BC	0.47 BC	13.7 CD	20.9 B	50.3 B
** MWCNTs 20/GA**_**3**_ **75 ppm composites**	34.8 A	9.4 A	14.8 A	1.7 A	1.0 A	2.8 A	0.47 BC	17.1 AB	26.3 A	78.6 A
** MWCNTs 30/GA**_**3**_ **75 ppm composites**	28.7 A-C	8.5 BC	12.6 B	1.1 B	0.7 B	1.9 BC	0.54 A-C	13.0 DE	20.5 B	50.1 B
** MWCNTs 40/GA**_**3**_ **75 ppm composites**	19.8 C	6.4 G	11.3 BC	0.9 B	0.6 B	1.5 C	0.55 A-C	6.4 IJ	12.3 D	34.6 E
** MWCNTs 50/GA**_**3**_ **75 ppm composites**	22.2 BC	6.6 FG	10.6 C	0.9 B	0.6 B	1.6 C	0.57 A-C	5.7 J	12.0 D	33.8 E
** LSD**_**0.05 (PS)**_	9.408	0.602	1.362	0.348	0.208	0.550	0.183	2.133	3.403	6.648

LSD_0.05_ = least significant differences at 0.05 probability. Means with the same letters in the same column are not significantly different (*P* ≤ 0.05) according to Tukey's test. Flower fresh weight “FW” (g), flower diameter “FD” (cm), vase life “VL” (days), chlorophyll a “Chlor. a” (mg/g FW), chlorophyll b “Chlor. b” (mg/g FW), total chlorophyll “Total Chlor.” (mg/g FW), carotenoid (mg/g FW), anthocyanin (mg/100g FW), total phenolic content “TPC” (mg GAE g^-1^) and 2,2-Diphenyl-1-picrylhydrazyl radical scavenging activity “DPPH” (%)

**Fig. 3:**
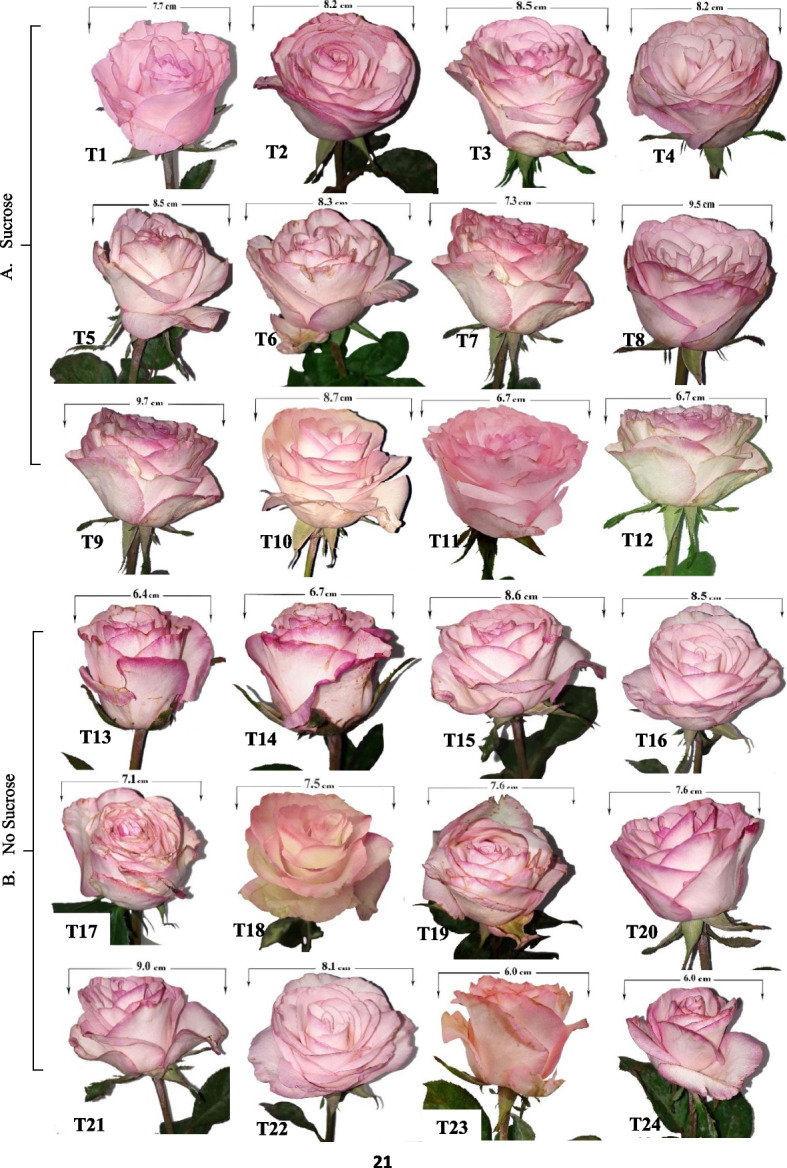
Clarifying the influence of MWCNTs or MWCNTs/ GA_3_ composites as a pulsing treatment; **A** with sucrose as holding solution on the maximum flower diameter as follows T1 = Control; distilled water, T2 = MWCNTs 10, T3 = MWCNTs 20, T4 = MWCNTs 30, T5 = MWCNTs 40, T6 = MWCNTs 50, T7 = GA_3_ 75, T8 = MWCNTs 10/GA_3_ 75 composites, T9 = MWCNTs 20/GA_3_ 75 composites, T10 = MWCNTs 30/GA_3_ 75 composites, T11 = MWCNTs 40/GA_3_ 75 composites, and T12 = MWCNTs 50/GA_3_ 75 ppm composites, and (**B**) with no sucrose as follows (T13 = Control; distilled water, T14 = MWCNTs 10, T15 = MWCNTs 20, T16 = MWCNTs 30, T17 = MWCNTs 40, T18 = MWCNTs 50, T19 = GA_3_ 75, T20 = MWCNTs 10/GA_3_ 75 composites, T21 = MWCNTs 20/GA_3_ 75 composites, T22 = MWCNTs 30/GA_3_ 75 composites, T23 = MWCNTs 40/GA_3_ 75 composites, and T24 = MWCNTs 50/GA_3_ 75 ppm composites, respectively, as illustrated

**Fig. 4:**
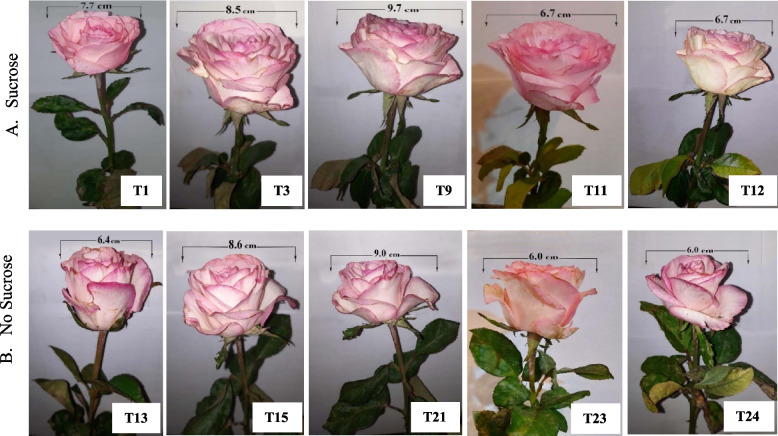
Contrasting the influence of MWCNTs or MWCNTs/ GA_3_ composites as a pulsing treatment with sucrose as holding solution on the morphological rose moonstone; flower diameter and stem quality **A**: (T1 = Control; distilled water, T3 = MWCNTs 20, T9 = MWCNTs 20/GA_3_ 75 composites, T11 = MWCNTs 40/GA_3_ 75 composites, and T12 = MWCNTs 50/GA_3_ 75 ppm composites) or with no sucrose as follows **B**: (T13 = Control; distilled water, T15 = MWCNTs 20, T21 = MWCNTs 20/GA_3_ 75 composites, T23 = MWCNTs 40/GA_3_ 75 composites, and T24 = MWCNTs 50/GA_3_ 75 ppm composites), respectively

According to Fig. 3A and B, the morphological characteristics of moonstone, maximum flower diameter, and stem quality preserved in different preservative solutions containing different concentrations varied from distinctive morphological characteristics when S was applied in the holding solution (Fig. 4A) combined with MWCNTs 20 ppm/GA_3_ 75 ppm composite (T9) or MWCNTs at 20 ppm (T3) as a novel anti-senescence agent engineered nanocomposites in pulsing solution if compared to the remaining treatments, particularly when NS (Fig. 4B) was applied to the holding solution with MWCNTs 50 (T24) or 40 ppm/GA_3_ 75 ppm composite (T23), as illustrated in (Figs. 3B and 4A and B).

#### Photosynthetic pigments; chlorophyll a, chlorophyll b, total chlorophyll, and carotenoid contents of cut fresh rose leaves of moonstone

Leaf yellowing caused by a reduction in green photosynthetic pigments, chlorophyll a, chlorophyll b, and total chlorophyll concentration, or an increase in carotenoid content is a typical sign of the end of vase life. Sucrose in holding solution significantly stimulates photosynthetic pigments; chlorophyll a 1.2 mg/g FW, chlorophyll b 0.7 mg/g FW, total chlorophyll 2.0 mg/g FW, and carotenoids 0.57 mg/g FW concentration of cut rose fresh leaves, respectively as presented in (Table 1) (Additional file 1: Table S2). There were significant differences in the main effect of the chlorophyll content between the MWCNT 20 ppm/GA_3_ 75 ppm composite and MWCNT 20 ppm alone as a pulsing solution. The chlorophyll a was 1.7 and 1.3 mg/g FW, chlorophyll b was 1.0 and 0.8 mg/g FW, while total chlorophyll was 2.8 and 2.2 mg/g FW, respectively, and there was a significant reduction in the carotenoid content of 0.47 and 0.51 mg/g FW, respectively (Table 1) (Additional file 1: Table S2).

**Fig. 5:**
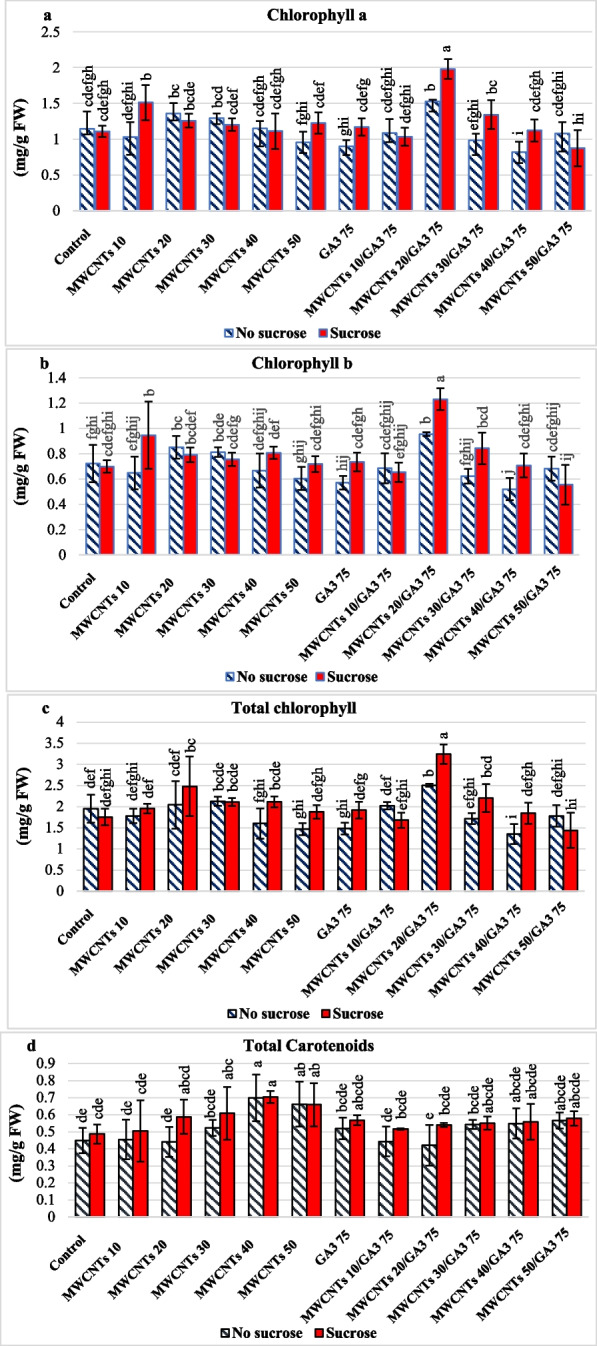
Cut rose moonstone photosynthetic pigment chlorophyll a (**a**), chlorophyll b (**b**), total chlorophyll (**c**) and total carotenoid (**d**) contents as a function of holding solution; with sucrose (S) or no sucrose (NS) and different pulsing solutions. The interactions are expressed as the means ± the standard error (SE). Bars with the same lowercase letters are not significantly different at the *P* < 0.05 level. The interactions are expressed as the means ± the standard error (SE). Statistics are provided in Additional file 1: Table S2

The highest significant values of chlorophyll a 1.9 mg/g FW, chlorophyll b 1.2 mg/g FW, and total chlorophyll 3.2 mg/g FW were recorded in the interaction between S in holding solution combined with MWCNTs 20 ppm/GA_3_ 75 ppm composite in pulsing solution, as shown in Additional file 1: Table S2 and (Fig. 5a, b, c). The minimum carotenoid content of 0.41 mg/g FW was observed when NS in the holding solution with MWCNTs 20 ppm/GA_3_ 75 ppm composite in the pulsing solution was applied, as shown in Additional file 1: Table S2 and Fig. 5d. According to Fig. 4B, the results showed that the highest carotenoid content (leaf yellowing) was revealed when NS was in holding solution with MWCNTs (50 or 40 ppm) alone or MWCNTs (50 or 40 ppm)/GA_3_ 75 ppm composite, as illustrated in Additional file 1: Table S2 and Fig. 5d.

#### Anthocyanin content in petals of fresh cut rose

**Fig. 6:**
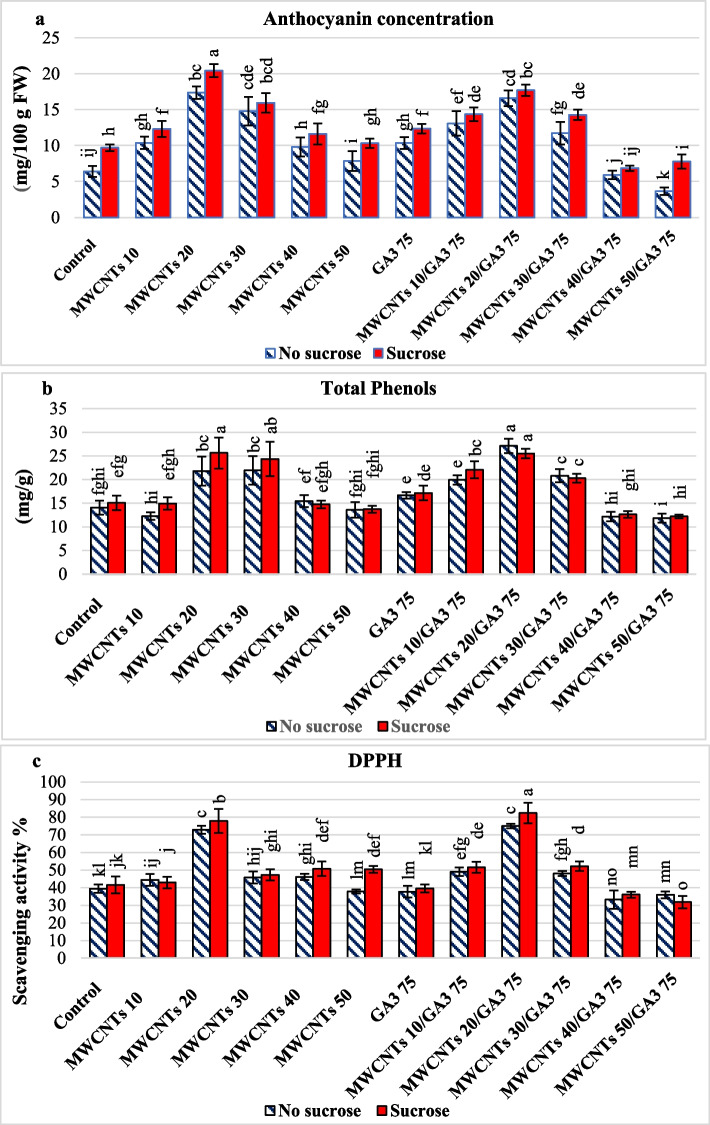
Cut rose moonstone anthocyanin concentration in fresh rose petals (**a**), total phenolic content (**b**), and DPPH radical scavenging activity (**c**) as a function of holding solution with sucrose (S) or no sucrose (NS) and different pulsing solutions. The interactions are expressed as the means ± the standard error (SE). Bars with the same lowercase letters are not significantly different at the *P* < 0.05 level; statistics are provided in Additional file 1: Table S3

Sucrose in the holding solution revealed a substantial increasing trend in the quantity of anthocyanin content (12.8 mg/100 g FW) of fresh cut rose petals, according to (Table 1) Additional file 1: Table S3 and Fig. 6a. When MWCNTs 20 ppm and MWCNTs 20 ppm/GA3 75 ppm composite were added to the pulsing solution, the anthocyanin content increased significantly to 18.9 and 17.1 mg/100 g FW, respectively. When applying the MWCNTs 50 or 40 ppm/GA3 75 ppm composite in pulsing solution, there was a substantial reduction in anthocyanin content of 5.7 and 6.4 mg/100 g FW respectively. According to Fig. 4 and as illustrated in Additional file 1: Table S3 and Fig. 6a, the highest anthocyanin content was reported when S in the holding solution was treated with MWCNTs at 20 ppm alone or MWCNTs 20 ppm/GA3 75 ppm composite in pulsing solution (20.4 and 17.7 mg/100 g FW, respectively). The lowest anthocyanin content 5.9 and 3.6 mg/100 g FW were observed when NS was applied in holding solution with MWCNTs (40 or 50 ppm)/GA_3_ 75 ppm composite in pulsing solution, respectively.

#### Total phenolic content (TPC) of the cut rose

According to Fig. 6b, the most important postharvest feature in moonstone roses is TPC in various preservative solutions, which reflects antioxidant activity. The highest value of TPC (27.1 mg GAE/g DW) was detected in the NS holding solution with MWCNTs (20 ppm)/GA_3_ 75 ppm composite in the pulsing solution; on the other hand, the lowest value (11.8 mg GAE/g DW) was observed in NS in the holding solution with MWCNTs (50 ppm)/GA_3_ 75 ppm composite in the pulsing solution as shown, in Additional file 1: Table S3.

#### DPPH radical scavenging activity of cut rose

Data about the DPPH radical scavenging activity of cut roses are shown in (Table 1) and Additional file 1: Table S3 and Fig. 6c. All treatments significantly affected the DPPH radical scavenging activity of the cut roses. Sucrose as a holding solution, MWCNTs (20 ppm)/GA_3_ 75 ppm composite, and MWCNTs alone at 20 ppm in the pulsing solution caused an increase in DPPH radical scavenging activity. The greatest DPPH radical scavenging percentages (82.3% and 77.8%) were achieved in roses treated by S as a holding solution with MWCNTs (20 ppm)/GA_3_ 75 ppm composite and MWCNTs alone at (20 ppm) in pulsing solution, respectively, and the lowest value (31.8%) was observed in S holding solution with MWCNTs (50 ppm)/GA_3_ 75 ppm composite in pulsing solution.

## Discussion

In this work, our results focused on the significant positive responses of all recorded quality parameters when moonstone fresh cut roses were treated with S compared with NS holding solution. Pragya et al. [28] and Young and Wan [43] reported that S provides a vital substrate for respiration as well as structural material and carbon skeletons for flower opening and mentioned that 2% S in the holding solution better regulated water uptake, water relations, and transpiration loss and improved the postharvest quality of cut roses, flower fresh weight, and flower opening and prolonged their vase life. It was found that S increased leaf photosynthetic pigments, chlorophyll a, chlorophyll b, total chlorophyll, and carotenoids, and the anthocyanin content of fresh cut rose also improved the impact of cytokinin on the delay of floral senescence and decreased the effect of ethylene on prolonging flower vase life [44, 45]. Our findings are in line with Bhawana et al. [46], Lama et al. [47], and Nguyen and Lim [48], who mentioned that S plays a role by enhancing antioxidant properties, such as increasing the antioxidant activity of 1, 1-diphenyl-2-picrylhydrazyl (DPPH) and reducing oxidative stress-mediated damage during rose flower senescence and the total phenolic content (TPC) of cut rose petals.

The morphologies of nanomaterials/nanoparticles vary depending on the desired use, with diameters ranging from 1 to 100 nm, size, dimension, origin, and composition: tubular, spherical, hollow, flat, conical, cylindrical, or irregular. NPs feature new and size-related physio-chemical characteristics that distinguish them from larger materials [9]. Several studies in horticulture and ornamental plants have indicated that CNTs are effective when used preharvest [6, 49], as they improve vegetative growth, flowering, and yield quality in carnations.

The results highlight the significant potencies of MWCNTs in low concentrations as a pulsing solution at 20 ppm associated with improving the postharvest quality of cut roses; increased flower fresh weight, flower opening, chlorophyll a, chlorophyll b, total chlorophyll, carotenoids, and anthocyanin; the antioxidant scavenging activity of 1, 1-diphenyl-2-picrylhydrazyl (DPPH); and reduced oxidative stress-mediated damage during rose flower senescence and total phenolic content (TPC) of fresh cut rose and prolonged vase life, whereas contrary results were observed in high MWCNT concentrations at 40 or 50 ppm. The same observations were reported by Ghorbanpour and Hadian [50] working on *Satureja khuzestanica* and Khodakovskaya et al. [51] on tobacco, as they mentioned that low doses of MWCNTs can increase cell development, while at greater doses, they significantly inhibit cell growth. It appears that the effects of MWCNTs are dose-dependent, as modest concentrations of MWCNTs exhibited growth-promoting effects with positive effects on physiology, biochemistry, cellular elongation [52, 53], cell size and xylem conducting tissue in *Catharanthus roseus* [54], xylem and phloem conducting tissues [53], nutrition, and water uptake [55], and photosynthesis efficiency [56]; however, high dosages caused significant drops that had an adverse effect on growth.

It was detected that the hollow morphology of MWCNTs can be identified even after GA_3_ adsorption, which ensures that the formation of the MWCNT/GA_3_ composite preserves the nanostructure. Additionally, the MWCNTs were dispersed evenly in water without aggregation. Given that the MWCNTs are delivered to the plant’s stem via water, it was critical to ensure that they were evenly distributed throughout the vase solution. Similar results were stated by Mousavi et al. [10] working on cut *Alstroemeria*.

Zahra and Rahim [23] indicated that a GA_3_ pulse treatment significantly improved the postharvest performance of cut rose flowers and leaves while also significantly reducing the typical increase in pH and conductivity of the cell sap. GA_3_ pulse treatment reduced levels of 1-aminocyclopropane-1-carboxylic acid (ACC) and ethylene production in rose [57]. Furthermore, the senescence and wilting-delaying effect of GA_3_ was connected to a reduction in the respiration rate [58, 59]. GA_3_ creates an inverse osmotic pressure in the cell and enhances water intake via starch and S hydrolysis in lilies and gladiolus [60]. Furthermore, the combination of GA_3_ pulsing solution treatment with S considerably improved the vase life of the cut rose flower when compared to treatment with S alone or the control [61]. Similar results were reported in our investigation since MWCNTs 20 ppm/GA_3_ 75 ppm composite, 75 ppm GA_3_, and MWCNTs 20 ppm with 20% S as pulsing solutions were substantially related to improving morpho-physiological characteristics postharvest quality and extending the life of cut rose flowers.

Our results indicated that the vase life can be affected by either MWCNTs or the MWCNTs/GA_3_ 75 ppm composite, depending on the concentration. MWCNTs at suitable concentrations (20 ppm) extend the vase life and quality by regulating the postharvest characteristics of cut rose. This finding aligns with recent findings showing the advantageous effects of MWCNTs on roses [62].

However, at high concentrations of MWCNTs (40 or 50 ppm), either MWCNTs alone or the MWCNTs/GA_3_ 75 ppm composite, the cut rose tips of the petals and leaves turned yellow, brown, and then black more quickly, affecting the quality characteristics and shortening the life of the cut flowers. This could be caused by MWCNTs accumulating within the xylem, which could have negative consequences, such as inhibiting nutrient, water, and plant hormone transport [10]. Di Zhang et al. [63] explained that the effect of MWCNTs on cut rose longevity was due to their high ability to be absorbed or transported by plant vascular tissue, which can cause increased accumulation within the xylem and vascular tissue blockage, resulting in toxicity, senescence, and wilting, especially at high doses. In agreement with our observations, Ghasempour et al. [54] mentioned that MWCNT treatments increased the activity of two major enzyme antioxidants, catalase and peroxidase. These findings support the concept that MWCNT treatment is associated with the activation of a defense system that provides plant resistance to unfavorable conditions.

Our method affirms that the hollow morphology of MWCNTs can be identified even after GA_3_ adsorption, which ensures that the formation of the MWCNT/GA_3_ composite as a novel engineered nanoparticle preserves the nanostructure, as previously described in both the Materials and Methods and Results sections. The high-resolution transmission electron microscopy (HR-TEM) of the MWCNTs/GA_3_ composite and FTIR spectra demonstrated that the MWCNTs/GA_3_ composite exhibits excellent dispersion properties in vase solution.

## Conclusion

The postharvest quality of cut rose flowers degrades; hence, enhancing the vase life quality is a vital step in guaranteeing the crop’s economic viability. This study sheds new light on the interaction between applying a solution containing MWCNTs with or without GA_3_ (MWCNTs/GA_3_ composites), which is a novel engineered nanoparticle as a pulsing and a holding solution with or without S to induce and improve the vase life and quality of cut *Rosa hybrida* cv. moonstone makes it easier for plants to absorb this nanotube. The current findings indisputably show that MWCNTs 20 ppm/GA_3_ 75 ppm composite or MWCNTs alone at 20 ppm in pulsing solution with S are superior and critical to the longevity, flower opening, and keeping quality of cut flowers by alleviating chlorophyll, carotenoids, and anthocyanin content, stimulating antioxidant defense such as total phenolic compounds and DPPH radical scavenging activity. The purpose of this study was to provide post-harvest advice for cut flowers to assist cut flower farmers. In order to create post-harvest management strategies, considerations regarding holding and pulsing solutions treatments for cut flowers should be included. Those materials act as a guideline for new post-harvest chemicals to be used in the production of commercial cut flowers. Additional studies examining the interactions of uniquely designed nanoparticle MWCNTs with plant hormones on cut flower systems are needed to understand their impacts on vase life, favorable physiological responses, negative effects, and optimal concentrations.

### Supplementary Information

**Supplementary Material 1.**

## Data Availability

This published paper and the supplementary data contain all the data created or analyzed during this investigation.
