# Supplementary material for: A novel efficient multi-walled carbon nanotubes/gibberellic acid composite for enhancement vase life and quality of Rosa hybrida cv. ‘Moonstone’
Source: BMC Plant Biol. 2024 Apr 3;24:239. doi: 10.1186/s12870-024-04925-9 (PMC10988866; doi:10.1186/s12870-024-04925-9)
Supplement: Supplementary file 1 — Supplementary Material 1. [file 12870_2024_4925_MOESM1_ESM.docx]

**Additional file 1:**

**Supplementary Tables S1.** Cut rose ʻMoonstoneʼ fresh weight, flower diameter, and vase life as a function of holding solution (HS); with sucrose (S) or no sucrose (NS), different pulsing solution (PS) with novel anti-senescence agent engineered nanocomposites and their interactions (HS x PS) (data in Fig.3 a, b and c).

| Main effect of **holding solution (HS)** with No Sucrose **(NS)** or with Sucrose **(S)** | | | | |
| --- | --- | --- | --- | --- |
| **Table S1** | | **Fresh weight (gm)** | **Flower diameter (cm)** | **Vase life (days)** |
| No Sucrose **(NS)** | | 24.6 B | 7.5 B | 12.0 B |
| Sucrose **(S) 20 gl-^1^** | | 27.2 A | 8.3 A | 12.4 A |
| Main effect of **Pulsing** **solution (PS)** with novel **anti-senescence agent** engineered nanocomposites | | | | |
| Distilled water (Control) | | 24.2 BC | 7.1 EF | 11.3 BC |
| MWCNTs 10 ppm | | 23.9 BC | 7.4 E | 12.1 B |
| MWCNTs 20 ppm | | 30.2 AB | 9.0 AB | 14.5 A |
| MWCNTs 30 ppm | | 28.0 A-C | 8.4 BC | 12.1 B |
| MWCNTs 40 ppm | | 25.0 BC | 8.0 CD | 11.6 BC |
| MWCNTs 50 ppm | | 22.2 BC | 8.1 CD | 11.3 BC |
| GA_3_ 75 ppm | | 24.2 BC | 7.6 DE | 11.8 BC |
| MWCNTs 10/GA_3_ 75 composites ppm | | 28.1 A-C | 8.8 B | 12.6 B |
| MWCNTs 20/GA_3_ 75 composites ppm | | 34.8 A | 9.4 A | 14.8 A |
| MWCNTs 30/GA_3_ 75 composites ppm | | 28.7 A-C | 8.5 BC | 12.6 B |
| MWCNTs 40/GA_3_ 75 composites ppm | | 19.8 C | 6.4 G | 11.3 BC |
| MWCNTs 50/GA_3_ 75 composites ppm | | 22.2 BC | 6.6 FG | 10.6 C |
| **Interaction between** **(HS x PS) holding solution and Pulsing solution** | | | | |
| **holding solution** | **Pulsing solution** |  |  |  |
| No Sucrose **(NS)** | T1= Distilled water (Control) | 24.1 d-g | 6.9 jk | 11.0 ef |
|  | T2= MWCNTs 10 ppm | 22.8 d-g | 6.8 k | 12.0 c-e |
|  | T3= MWCNTs 20 ppm | 30.1 a-d | 8.6 c-e | 14.6 ab |
|  | T4= MWCNTs 30 ppm | 27.9 b-f | 8.2 e-g | 11.6 c-e |
|  | T5= MWCNTs 40 ppm | 21.2 e-g | 7.7 hi | 11.6 c-e |
|  | T6= MWCNTs 50 ppm | 20.7 fg | 7.7 g-i | 11.3 d-f |
|  | T7= GA_3_ 75 ppm | 25.0 d-g | 7.4 ij | 11.6 c-e |
|  | T8= MWCNTs 10/GA_3_ 75 composites ppm | 28.5 a-e | 8.4 d-f | 12.6 c |
|  | T9= MWCNTs 20/GA_3_ 75 composites ppm | 33.9 ab | 8.7 cd | 14.0 b |
|  | T10= MWCNTs 30/GA_3_ 75 composites ppm | 24.5 d-g | 8.1 e-h | 12.6 c |
|  | T11= MWCNTs 40/GA_3_ 75 composites ppm | 18.5 g | 6.1 l | 11.3 d-f |
|  | T12= MWCNTs 50/GA_3_ 75 composites ppm | 18.7 g | 6.2 l | 10.3 f |
| Sucrose **(S)**  **20 gl-^1^** | T13= Distilled water (Control) | 24.3 d-g | 7.3 ij | 11.6 c-e |
|  | T14= MWCNTs 10 ppm | 25.0 d-g | 8.0 f-h | 12.3 cd |
|  | T15= MWCNTs 20 ppm | 30.3 a-d | 9.4 b | 14.3 b |
|  | T16= MWCNTs 30 ppm | 28.1 a-f | 8.7 cd | 12.6 c |
|  | T17= MWCNTs 40 ppm | 28.8 a-e | 8.4 d-f | 11.6 c-e |
|  | T18= MWCNTs 50 ppm | 23.8 d-g | 8.4 d-f | 11.3 d-f |
|  | T19= GA_3_ 75 ppm | 23.5 d-g | 7.9 gh | 12.0 c-e |
|  | T20= MWCNTs 10/GA_3_ 75 composites ppm | 27.7 b-f | 9.2 b | 12.6 c |
|  | T21= MWCNTs 20/GA_3_ 75 composites ppm | 35.7 a | 10.2 a | 15.6 a |
|  | T22= MWCNTs 30/GA_3_ 75 composites ppm | 32.9 a-c | 8.9 bc | 12.6 c |
|  | T23= MWCNTs 40/GA_3_ 75 composites ppm | 21.2 e-g | 6.7 k | 11.3 d-f |
|  | T24= MWCNTs 50/GA_3_ 75 composites ppm | 25.7 c-g | 7.1 jk | 11.0 ef |
| **LSD_0.05 (HS)_** | | 2.249 | 0.144 | 0.326 |
| **LSD_0.05 (PS)_** | | 9.4077 | 0.602 | 1.362 |
| **LSD_0.05_ for (HS x PS)** | | 7.75 | 0.495 | 1.122 |

LSD_0.05_ = least significant differences at 0.05 probability.

Means with the same letter are not significantly different (P ≤ 0.05) according to Tukey's test.

**Supplementary Tables S2.** Cut rose fresh leaves ʻMoonstoneʼ chlorophyll a, chlorophyll b, total chlorophyll and carotenoid contents as a function of holding solution (HS); with sucrose (S) or no sucrose (NS), different pulsing solution (PS) with novel anti-senescence agent engineered nanocomposites and their interactions (HS x PS) (data in Fig.4 a, b, c and d).

| Main effect of **holding solution (HS)** with No Sucrose **(NS)** or with Sucrose **(S)** | | | | | |
| --- | --- | --- | --- | --- | --- |
| **Table S2** | | **chlorophyll a (mg/g FW)** | **chlorophyll b (mg/g FW)** | **total chlorophyll (mg/g FW)** | **carotenoid contents (mg/g FW)** |
| No Sucrose **(NS)** | | 1.1 B | 0.6 B | 1.8 B | 0.52 B |
| Sucrose **(S)** | | 1.2 A | 0.7 A | 2.0 A | 0.57 A |
| Main effect of **Pulsing solution (PS)** with novel **anti-senescence agent** engineered nanocomposites | | | | | |
| Distilled water (Control) | | 1.1 B | 0.7 B | 1.8 BC | 0.46 C |
| MWCNTs 10 ppm | | 1.2 B | 0.7 B | 1.8 BC | 0.47 BC |
| MWCNTs 20 ppm | | 1.3 B | 0.8 B | 2.2 B | 0.51 BC |
| MWCNTs 30 ppm | | 1.2 B | 0.7 B | 2.1BC | 0.56 A-C |
| MWCNTs 40 ppm | | 1.1 B | 0.7 B | 1.8 BC | 0.69 A |
| MWCNTs 50 ppm | | 1.0 B | 0.6 B | 1.6 C | 0.65 AB |
| GA_3_ 75 ppm | | 1.0 B | 0.6 B | 1.6 C | 0.54 A-C |
| MWCNTs 10/GA_3_ 75 composites ppm | | 1.0 B | 0.6 B | 1.8 BC | 0.47 BC |
| MWCNTs 20/GA_3_ 75 composites ppm | | 1.7 A | 1.0 A | 2.8 A | 0.47 BC |
| MWCNTs 30/GA_3_ 75 composites ppm | | 1.1 B | 0.7 B | 1.9 BC | 0.54 A-C |
| MWCNTs 40/GA_3_ 75 composites ppm | | 0.9 B | 0.6 B | 1.5 C | 0.55 A-C |
| MWCNTs 50/GA_3_ 75 composites ppm | | 0.9 B | 0.6 B | 1.6 C | 0.57 A-C |
| **Interaction between (HS x PS) holding solution and Pulsing solution** | | | | | |
| **holding solution** | **Pulsing solution** |  |  |  |  |
| No Sucrose **(NS)** | Distilled water (Control) | 1.1 c-h | 0.7 f-i | 1.9 d-f | 0.44 de |
|  | MWCNTs 10 ppm | 1.0 d-i | 0.6 e-j | 1.7 d-i | 0.45 de |
|  | MWCNTs 20 ppm | 1.3 bc | 0.8 bc | 2.0 c-f | 0.43 de |
|  | MWCNTs 30 ppm | 1.2 b-d | 0.8 b-e | 2.1 b-e | 0.52 b-e |
|  | MWCNTs 40 ppm | 1.1 c-h | 0.6 d-j | 1.5 f-i | 0.69 a |
|  | MWCNTs 50 ppm | 0.9 f-i | 0.6 g-j | 1.4 g-i | 0.66 ab |
|  | GA_3_ 75 ppm | 0.8 g-i | 0.5 h-j | 1.4 g-i | 0.51 b-e |
|  | MWCNTs 10/GA_3_ 75 composites ppm | 1.0 c-i | 0.6 c-j | 2.0 d-f | 0.44 de |
|  | MWCNTs 20/GA_3_ 75 composites ppm | 1.5 b | 0.9 b | 2.4 b | 0.41 e |
|  | MWCNTs 30/GA_3_ 75 composites ppm | 0.9 e-i | 0.6 f-j | 1.7 e-i | 0.54 b-e |
|  | MWCNTs 40/GA_3_ 75 composites ppm | 0.8 i | 0.5 j | 1.3 i | 0.54 a-e |
|  | MWCNTs 50/GA_3_ 75 composites ppm | 1.0 c-i | 0.6 c-j | 1.7 d-i | 0.56 a-e |
| Sucrose **(S)** | Distilled water (Control) | 1.1 c-h | 0.6 c-i | 1.7 d-i | 0.48 c-e |
|  | MWCNTs 10 ppm | 1.5 b | 0.9 b | 1.9 d-f | 0.50 c-e |
|  | MWCNTs 20 ppm | 1.2 b-e | 0.7 b-f | 2.4 bc | 0.58 a-d |
|  | MWCNTs 30 ppm | 1.2 c-f | 0.7 c-g | 2.1 b-e | 0.60 a-c |
|  | MWCNTs 40 ppm | 1.1 c-h | 0.8 d-f | 2.1 b-e | 0.70 a |
|  | MWCNTs 50 ppm | 1.2 c-f | 0.7 c-i | 1.8 d-h | 0.65 ab |
|  | GA_3_ 75 ppm | 1.1 c-g | 0.7 c-h | 1.9 d-g | 0.56 a-e |
|  | MWCNTs 10/GA_3_ 75 composites ppm | 1.0 d-i | 0.6 e-j | 1.6 e-i | 0.51 b-e |
|  | MWCNTs 20/GA_3_ 75 composites ppm | 1.9 a | 1.2 a | 3.2 a | 0.53 b-e |
|  | MWCNTs 30/GA_3_ 75 composites ppm | 1.3 bc | 0.8 b-d | 2.1 b-d | 0.54 a-e |
|  | MWCNTs 40/GA_3_ 75 composites ppm | 1.1 c-h | 0.7 c-i | 1.8 d-h | 0.55 a-e |
|  | MWCNTs 50/GA_3_ 75 composites ppm | 0.8 hi | 0.5 ij | 1.4 hi | 0.57 a-e |
| **LSD_0.05 (HS)_** | | **0.083** | **0.049** | **0.132** | **0.044** |
| **LSD_0.05 (PS)_** | | **0.348** | **0.208** | **0.550** | **0.183** |
| **LSD_0.05_ for (HS x PS)** | | **0.286** | **0.1712** | **0.4532** | **0.1506** |

LSD_0.05_ = least significant differences at 0.05 probability.

Means with the same letter are not significantly different (P ≤ 0.05) according to Tukey's test.

**Supplementary Tables S3.** Cut rose ʻMoonstoneʼ anthocyanin concentration in fresh rose petals, total phenolic content (TPC), and DPPH radical scavenging activity as a function of holding solution (HS); with sucrose (S) or no sucrose (NS), different pulsing solution (PS) with novel anti-senescence agent engineered nanocomposites and their interactions (HS x PS) (data in Fig.5 a, b and c).

| Main effect of **holding solution (HS)** with No Sucrose **(NS)** or with Sucrose **(S)** | | | | |
| --- | --- | --- | --- | --- |
| **Table S3** | | **anthocyanin**  **(mg/100g FW)** | **TPC**  **(mg GAE g^-1^)** | **DPPH**  **(%)** |
| No Sucrose **(NS)** | | 10.6 B | 17.2 B | 47.1 B |
| Sucrose **(S)** | | 12.8 A | 18.1 A | 50.3 A |
| Main effect of **Pulsing solution (PS)** with novel **anti-senescence agent** engineered nanocomposites | | | | |
| Distilled water (Control) | | 8.0 HI | 14.5 CD | 40.4 CDE |
| MWCNTs 10 ppm | | 11.3 EF | 13.5 CD | 43.6 BCD |
| MWCNTs 20 ppm | | 18.9 A | 23 AB | 75.3 A |
| MWCNTs 30 ppm | | 15.3 BC | 23 AB | 46.4 BC |
| MWCNTs 40 ppm | | 10.7 FG | 15.0 CD | 48.3 B |
| MWCNTs 50 ppm | | 9.0 GH | 13.6 CD | 44.1 BCD |
| GA_3_ 75 ppm | | 11.3 EF | 16.9 C | 38.6 DE |
| MWCNTs 10/GA_3_ 75 composites ppm | | 13.7 CD | 20.9 B | 50.3 B |
| MWCNTs 20/GA_3_ 75 composites ppm | | 17.1 AB | 26.3 A | 78.6 A |
| MWCNTs 30/GA_3_ 75 composites ppm | | 13.0 DE | 20.5 B | 50.1 B |
| MWCNTs 40/GA_3_ 75 composites ppm | | 6.4 IJ | 12.3 D | 34.6 E |
| MWCNTs 50/GA_3_ 75 composites ppm | | 5.7 J | 12.0 D | 33.8 E |
| **Main effect of interaction between (HS x PS) holding solution and Pulsing solution** | | | | |
| **holding solution** | **Pulsing solution** |  |  |  |
| No Sucrose **(NS)** | Distilled water (Control) | 6.4 ij | 14.0 f-i | 39.4 kl |
|  | MWCNTs 10 ppm | 10.4 gh | 12.2 hi | 44.4 ij |
|  | MWCNTs 20 ppm | 17.3 bc | 21.7 bc | 72.8 c |
|  | MWCNTs 30 ppm | 14.8 c-e | 21.9 bc | 45.7 h-j |
|  | MWCNTs 40 ppm | 9.8 h | 15.4 ef | 46.1 g-i |
|  | MWCNTs 50 ppm | 7.8 i | 13.5 f-i | 37.8 lm |
|  | GA_3_ 75 ppm | 10.3 gh | 16.6 e | 37.7 lm |
|  | MWCNTs 10/GA_3_ 75 composites ppm | 13.1 ef | 19.9 e | 49.0 e-g |
|  | MWCNTs 20/GA_3_ 75 composites ppm | 16.6 cd | 27.12 a | 74.9 c |
|  | MWCNTs 30/GA_3_ 75 composites ppm | 11.7 fg | 20.8 c | 48.0 f-h |
|  | MWCNTs 40/GA_3_ 75 composites ppm | 5.9 j | 12.1 hi | 33.2 no |
|  | MWCNTs 50/GA_3_ 75 composites ppm | 3.6 k | 11.8 i | 35.8 mn |
| Sucrose **(S)** | Distilled water (Control) | 9.7 h | 15.0 e-g | 41.5 jk |
|  | MWCNTs 10 ppm | 12.3 f | 14.9 e-h | 42.9 j |
|  | MWCNTs 20 ppm | 20.4 a | 25.6 a | 77.8 b |
|  | MWCNTs 30 ppm | 15.9 b-d | 24.3 ab | 47.2 g-i |
|  | MWCNTs 40 ppm | 11.6 fg | 14.7 e-h | 50.6 d-f |
|  | MWCNTs 50 ppm | 10.3 gh | 13.7 f-i | 50.4 d-f |
|  | GA_3_ 75 ppm | 12.3 f | 17.1 de | 39.4 kl |
|  | MWCNTs 10/GA_3_ 75 composites ppm | 14.3 de | 22.0 bc | 51.5 de |
|  | MWCNTs 20/GA_3_ 75 composites ppm | 17.7 bc | 25.5 a | 82.3 a |
|  | MWCNTs 30/GA_3_ 75 composites ppm | 14.3 de | 20.2 c | 52.1 d |
|  | MWCNTs 40/GA_3_ 75 composites ppm | 6.8 ij | 12.6 g-i | 36.0 mn |
|  | MWCNTs 50/GA_3_ 75 composites ppm | 7.8 i | 12.1 hi | 31.8 o |
| **LSD_0.05 (HS)_** | | **0.509** | **0.813** | **1.589** |
| **LSD_0.05 (PS)_** | | **2.133** | **3.403** | **6.648** |
| **LSD_0.05_ for (HS x PS)** | | **1.756** | **2.803** | **2.803** |

**LSD_0.05_** = least significant differences at 0.05 probability; Means with the same letter are not significantly different (P ≤ 0.05) according to Tukey's test; GAE: gallic acid equivalent

**Abbreviations**

Carot.: carotenoid; Chl. a: Chlorophyll a; Chl. b: Chlorophyll b; DPPH: 2,2-Diphenyl-1-picrylhydrazyl; FAE: Ferulic acid equivalent; FCR: Folin-Ciocalteau reagent; FTIR: Fourier transform infrared; GA_3:_ Gibberellic acid; GAE: Gallic acid equivalent; HR-TEM: High-resolution transmission electron microscopy; MWCNTs/GA_3_ 75 composites: Multi-walled carbon nanotubes/gibberellic acid 75 ppm composites as a novel nanocomposite; MWCNTs: Multi-walled carbon nanotubes; Total Chls: Total chlorophyll; TPC: Total phenolic content; PGRs: Plant growth regulators.
